# Fosinopril inhibits Ang II-induced VSMC proliferation, phenotype transformation, migration, and oxidative stress through the TGF-β1/Smad signaling pathway

**DOI:** 10.1515/biol-2022-0955

**Published:** 2024-11-22

**Authors:** Siqi Chen, Lingxiang Ye

**Affiliations:** Department of Electrocardiogram Diagnosis, Zhejiang Hospital, Hangzhou, Zhejiang, 310013, China

**Keywords:** vascular remodeling, VSMC, fosinopril, Ang II, oxidative stress, hypertension

## Abstract

Fosinopril (FOS) is an angiotensin-converting enzyme inhibitor that can decrease angiotensin II (Ang II) formation, thereby reducing systemic vasoconstriction. This study investigated the impact of FOS on vascular smooth muscle cell (VSMC) phenotypic transformation in hypertension. Experiments using western blotting revealed that FOS inhibits the Ang II-induced downregulation of α-SMA and SM22α and the upregulation of OPN in VSMCs. In addition, CCK8 assays, EdU staining, and Transwell assays demonstrated that FOS reduces Ang II-induced increases in VSMC cell viability, proliferation, migration, and MMP2 and MMP9 expression. Moreover, immunofluorescence and ELISA experiments showed that FOS suppresses Ang II-induced increases in ROS levels, NAD(P)H activity, and NOX2 and NOX4 expression in VSMCs. Western blotting also indicated that FOS inhibits Ang II-induced increases in TGF-β1 and p-Smad2/3 expression in VSMCs. Finally, FOS mitigates Ang II-induced VSMC proliferation, phenotypic transformation, migration, and oxidative stress by inhibiting the TGF-β1/Smad signaling pathway. In conclusion, these results suggest that FOS could be effective in managing vascular diseases, including hypertension.

## 1 Introduction

Hypertension is a prevalent non-communicable disease affecting over one-third of the global population. Inadequate management of hypertension can lead to severe myocardial and coronary artery damage [1]. As its incidence rises annually, its recognition as a significant risk factor for cardiovascular disease continues to grow [2]. While lifestyle changes and pharmacological treatments are essential for preventing and managing hypertension, their overall efficacy remains limited, and hypertension-related costs account for 6.61% of the medical budget [3]. Thus, more comprehensive investigations into the pathogenesis and pharmacological management of hypertension are warranted.

Vascular smooth muscle cells (VSMCs) constitute the arterial matrix and have been found to play essential roles in vascular disease [4]. They can repeatedly alternate between contractile and synthetic phenotypes to adapt to external changes, and their synthetic phenotype is characterized by increased proliferation and migration [5,6]. This phenotypic transformation is considered a key process in vascular remodeling associated with hypertension. Angiotensin II (Ang II), a primary mediator of hypertension, regulates vasoconstriction and contributes to VSMC enlargement [7]. Previous studies have shown that Ang II-induced VSMC phenotypic transformation can contribute to hypertension [8]. Based on these considerations, we used an *in vitro* hypertension model in our present study by administering Ang II.

Fosinopril (FOS), an angiotensin-converting enzyme (ACE) inhibitor, binds competitively to ACE, reducing Ang II formation and thereby mitigating systemic vasoconstriction [9]. Research has shown that its anti-atherosclerotic effects are not solely due to blood pressure reduction but also to Ang II inhibition [10]. FOS, with its dual and compensatory excretion pathways, may also be beneficial in treating heart failure [11]. However, the specific effects and mechanisms of FOS on VSMCs remain unclear and require further investigation.

In this study, it was found that FOS inhibits Ang II-induced VSMC proliferation, phenotype transformation, migration, and oxidative stress through the TGF-β1/Smad signaling pathway.

## 2 Method

### 2.1 Cell culture

Primary human VSMCs (CRL-1999) were purchased from the American Type Culture Collection and cultured in DMEM (Sigma-Aldrich, USA) supplemented with 10% fetal bovine serum (Gibco, MA, USA). The cells were maintained in a humidified incubator at 37°C with 95% air and 5% CO_2_.

The experiment was divided into three groups: (1) the control group, which underwent no treatment; (2) the Ang II group, where VSMCs were incubated with 1 μM Ang II (Calbiochem, CA, USA) for 24 h; and (3) the Ang II + Fos group, where cells were first treated with 1 μM Ang II and then with 5 μM FOS (Fos, Calbiochem, CA, USA) for 2 h, following the Ang II treatment.

### 2.2 Cell counting kit 8

VSMCs were seeded at a density of 1 × 10^4^ cells per well in a 96-well plate. Following cell adhesion, they were subjected to the experimental treatments. After the appropriate incubation period, CCK-8 reagent (Sigma-Aldrich, USA) was added to each well, and the absorbance was measured at 450 nm using a microplate reader after 2 h.

### 2.3 EdU incorporation assay

VSMCs were seeded on glass slides and incubated with 5-ethynyl-2-deoxyuridine (EdU, RiboBio, R11053.2, Guangzhou, China) for 12 h. They were then fixed, permeabilized, and treated with the Apollo^®^ reaction mixture for 30 min in the dark. Next, they were counterstained, and the cells were observed using a laser scanning confocal microscope (Leica SP8).

### 2.4 Cell migration assay

VSMCs were plated in the upper chamber of a 24-well Transwell plate (Corning, New York, USA) at a density of 1 × 10^5^ cells per well. The lower chamber contained 20 ng/mL platelet-derived growth factor-BB (PDGF-BB, PeproTech, USA). Migrated cells were fixed with 4% paraformaldehyde and stained with crystal violet (Beyotime, Nanjing, China). The stained cells were then examined under a microscope.

### 2.5 Measurement of reactive oxygen species (ROS)

For this experiment, ROS levels were assessed using the DCFH-DA (Beyotime Biotechnology, Nanjing, China) ROS detection kit. VSMCs were treated with 10 μM DCFH-DA for 20 min in the dark, and the ROS levels were quantified using imaging with a confocal laser microscope.

## 3 Enzyme-linked immunosorbent assay (ELISA)

The level of NAD(P)H oxidase was measured using an ELISA kit (MultiSciences, Hangzhou, China) according to the manufacturer’s instructions.

### 3.1 Immunofluorescence

Here, the cells were washed five times with phosphate-buffered saline (PBS), fixed with 4% paraformaldehyde for 10 min, permeabilized with 0.3% Triton X-100 for 10 min, and blocked with BSA for 1 h. Primary antibodies were applied overnight at 4°C, followed by PBS washing. The cells were then stained with 4′,6-diamidino-2-phenylindole, and fluorescent secondary antibodies. Images were acquired using a laser confocal microscope.

### 3.2 Western blotting

Cellular proteins were extracted using a lysis buffer, and protein concentrations were determined with a BCA kit (Beyotime, Nanjing, China). Proteins were separated by electrophoresis, transferred to a polyvinylidene fluoride membrane, and blocked with 5% skim milk for 1 h. After washing, the membrane was incubated overnight at 4°C with primary antibodies. Following additional washing, the membrane was incubated with secondary antibodies (1:1,000, Cell Signaling Technology) for 1 h at room temperature. Chemiluminescent signals were detected using the BeyoECL Star Kit (Beyotime, Nanjing, China), and band intensities were analyzed with ImageJ 1.43 software.

### 3.3 Statistical analysis

Data analysis was conducted using GraphPad Prism V8.0 software. One-way analysis of variance was used for comparisons between multiple groups, while an unpaired Student’s *t*-test was employed for comparisons between two groups. Each experiment was performed in triplicate, and *p* < 0.05 was considered statistically significant.

## 4 Results

### 4.1 FOS inhibits Ang II-induced phenotypic transformation of VSMCs

**Figure 1:**
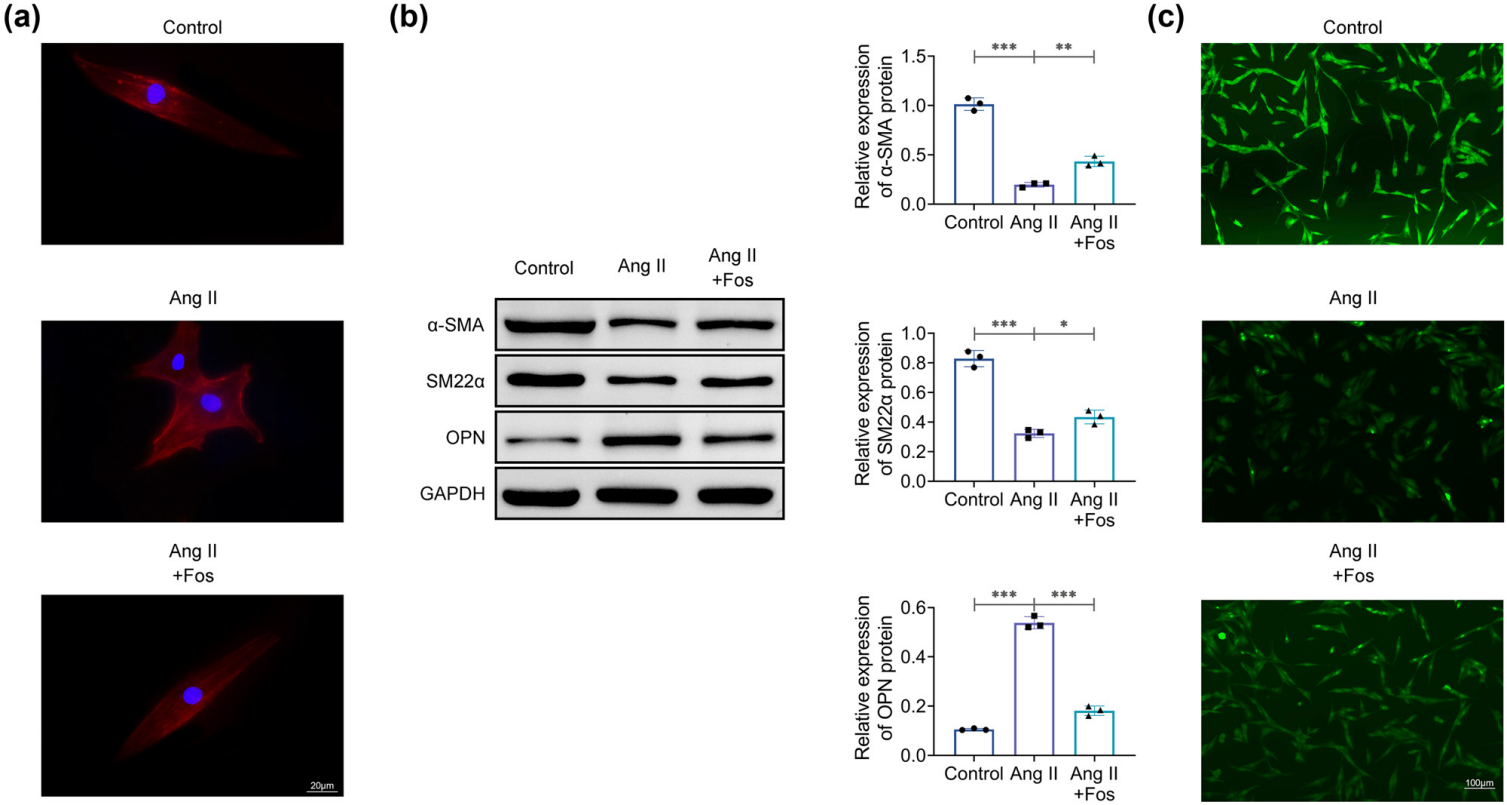
Fosinopril inhibits Ang II-induced phenotypic transformation of VSMCs. (a) Microscopic images showing VSMC morphology. (b) Western blot analysis of protein levels for α-SMA, SM22α, and OPN. (c) Immunofluorescence staining of SM22α. Data are presented as mean ± SD. *p < 0.05, **p < 0.01, and ***p < 0.001 compared to the control/Ang II group. *n* = 3.

After treating VSMCs with Ang II, the cells were found to exhibit increased OPN expression and decreased levels of the contractile proteins α-SMA and SM22α compared to the control group (Figure 1). This finding indicates a phenotypic transition of VSMCs from a contractile to a synthetic state. In addition, the administration of FOS was found to reverse these changes, demonstrating that Ang II successfully induces VSMC phenotypic transformation and that FOS inhibits this transformation (Figure 1).

### 4.2 FOS inhibits Ang II-induced VSMC proliferation

**Figure 2:**
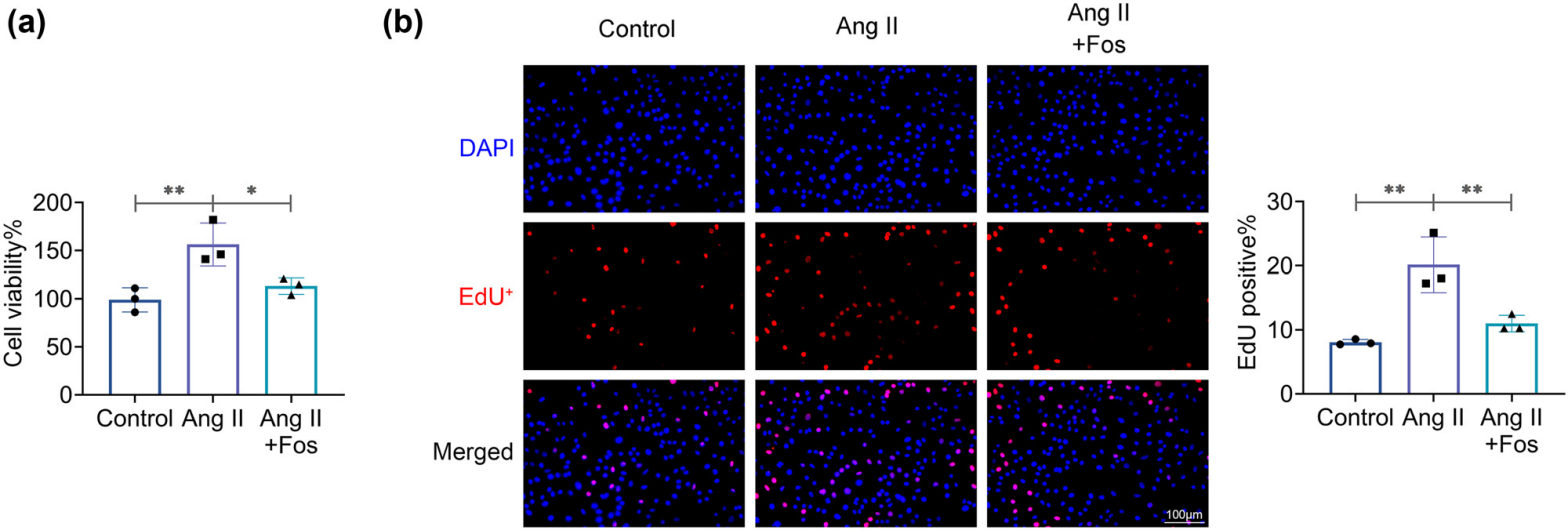
Fosinopril inhibits Ang II-induced VSMC proliferation. (a) Cell viability was assessed using the CCK-8 assay. (b) Representative images from the EdU incorporation assay and the percentage of EdU-positive cells. Data are presented as mean ± SD. *p < 0.05 and **p < 0.01 compared to the control/Ang II group. *n* = 3.

CCK8 and EdU assays were conducted to assess the impact of FOS on VSMC proliferation. As shown in Figure 2a and b, VSMC activity and proliferation were significantly higher in the Ang II group compared to the control group. In addition, these effects could be reversed by FOS treatment, indicating that FOS effectively inhibits Ang II-induced VSMC proliferation.

### 4.3 FOS inhibits Ang II-induced VSMC migration

**Figure 3:**
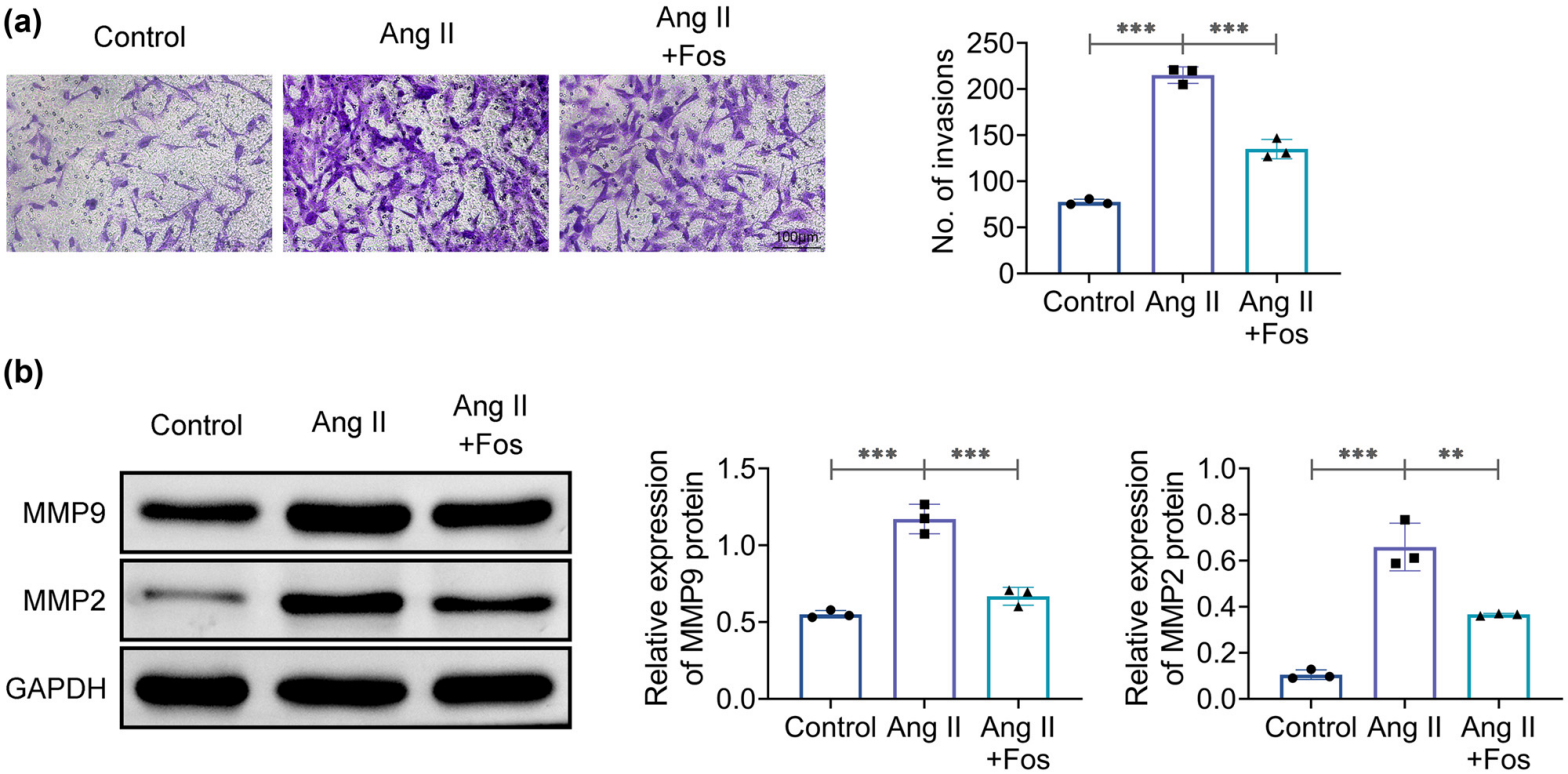
Fosinopril inhibits Ang II-induced VSMC migration. (a) Representative images of the Transwell assay and quantification of migrating cells. (b) Western blot analysis of MMP2 and MMP9 protein levels. Data are presented as mean ± SD. **p < 0.01 and ***p < 0.001 compared to the control/Ang II group. *n* = 3.

Matrix metalloproteinases (MMPs) are responsible for the proteolytic degradation of extracellular matrix proteins and play a role in endothelial cell migration [12]. To evaluate the effects of FOS on VSMC migration, Transwell chamber assays and western blotting were performed. The results showed that Ang II treatment led to increased VSMC migration and elevated MMP2 and MMP9 expression compared to the control group (Figure 3a and b). Furthermore, these effects could be reversed following FOS administration (Figure 3a and b), suggesting that FOS inhibits Ang II-induced VSMC migration.

### 4.4 FOS inhibits Ang II-induced oxidative stress in VSMCs

**Figure 4:**
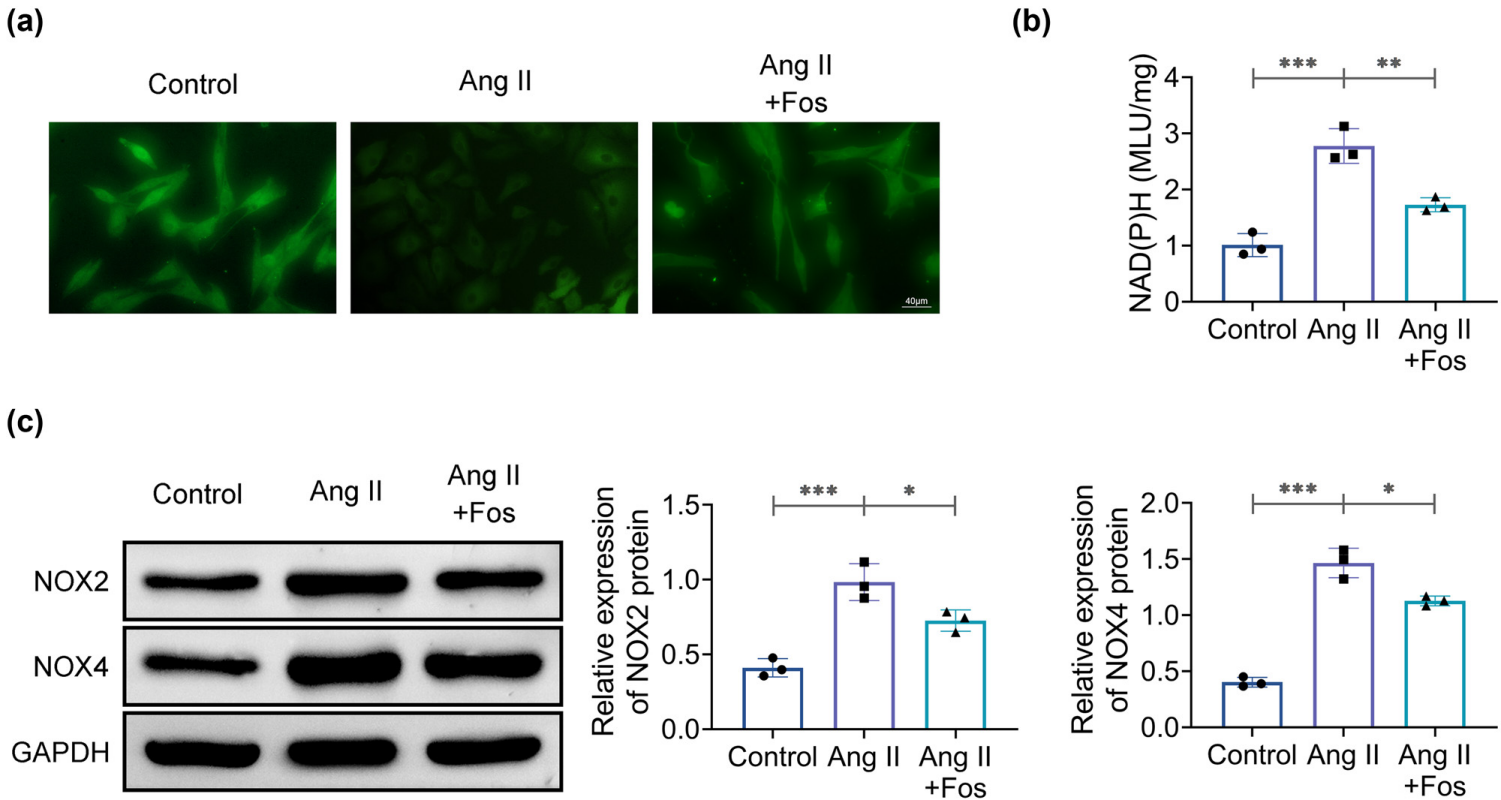
Fosinopril inhibits oxidative stress in VSMCs. (a) Immunofluorescence detection of ROS levels. (b) NAD(P)H oxidase activity measured by ELISA. (c) Western blot analysis of NOX2 and NOX4 protein levels. Data are presented as mean ± SD. *p < 0.05, **p < 0.01, and ***p < 0.001 compared to the control/Ang II group. *n* = 3.

Next, the levels of ROS and the expression of NOX2 and NOX4 in VSMCs were measured. We found that Ang II treatment resulted in increased ROS levels and NOX2 activity, as well as elevated NOX2 and NOX4 expression compared to the control group (Figure 4a–c, middle). Moreover, these changes were reversed following FOS administration (Figure 4a–c, right), indicating that FOS can inhibit Ang II-induced oxidative stress in VSMCs.

### 4.5 FOS inhibits the TGF-β1/Smad pathway

**Figure 5:**
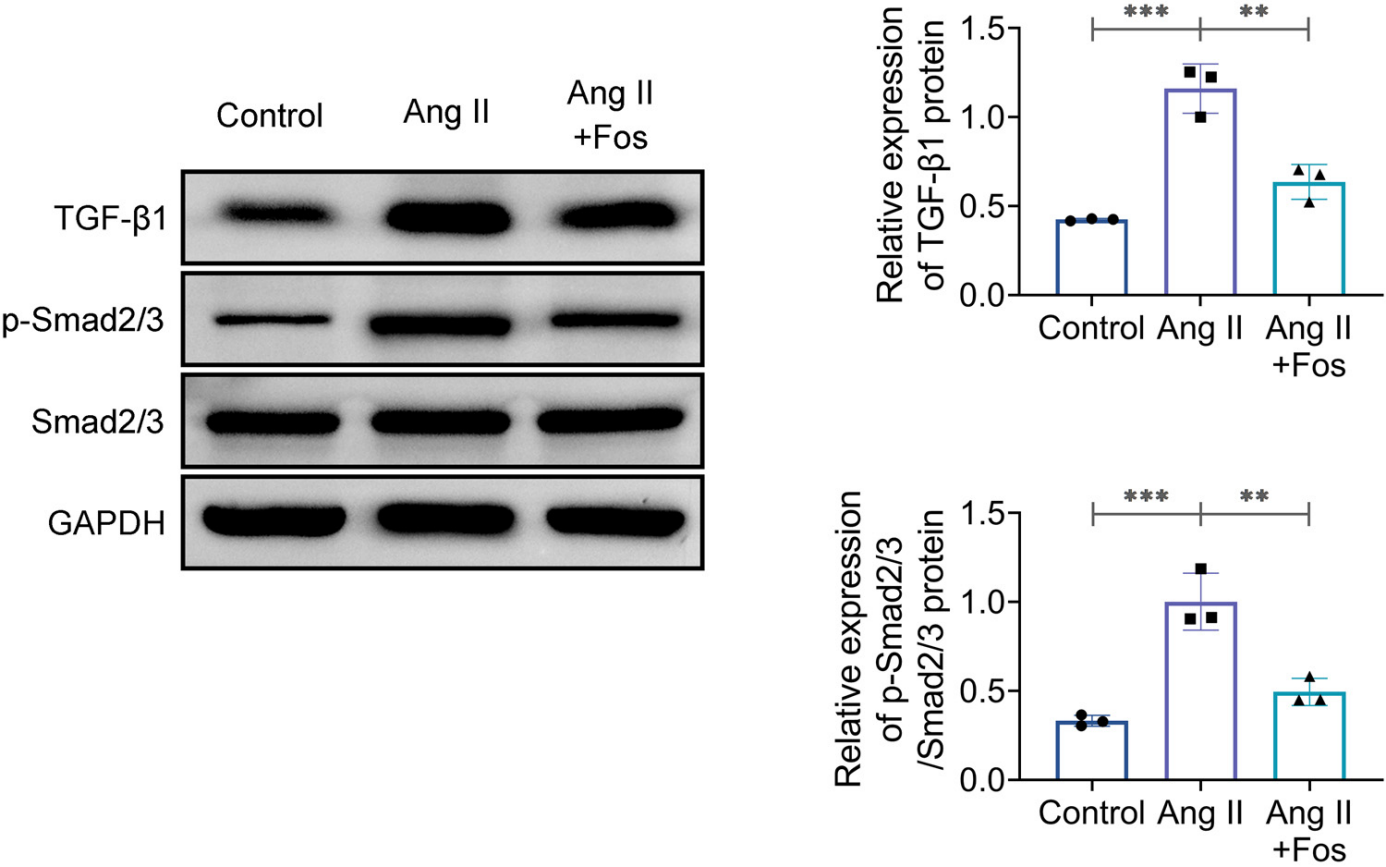
Fosinopril inhibits the TGF-β1/Smad signaling pathway. Western blot analysis of TGF-β1, Smad2, and p-Smad2/3 protein levels. Data are presented as mean ± SD. **p < 0.01 and ***p < 0.001 compared to the control/Ang II group. *n* = 3.

Finally, the effects of FOS on the TGF-β1/Smad signaling pathway in VSMCs were investigated. Ang II treatment led to increased expression of TGF-β1 and p-Smad2/3 compared to the control group (Figure 5, middle). This effect was reversed by FOS treatment (Figure 5, right), demonstrating that FOS inhibits the TGF-β1/Smad pathway in VSMCs.

## 5 Discussion

In this present study, we experimented on an Ang II-induced VSMC phenotypic transformation model and observed that, in the Ang II group, contractile proteins in VSMCs were downregulated, while synthetic proteins were upregulated, accompanied by increased migration, proliferation, and oxidative stress. Moreover, treatment using FOS could mitigate these effects induced by Ang II by targeting the TGF-β1/Smad signaling pathway.

Vascular remodeling is influenced by the external environment, and when it exceeds the adaptive capacity, it can lead to hypertension or other complications [13]. Then, the maladaptive vascular remodeling results in phenotypic alterations of VSMCs, which can contribute to hypertension [14]. Elevated serum levels of Ang II have been observed in patients with hypertension. Ang II has been shown to induce VSMC phenotypic transformation, with synthetic VSMCs exhibiting increased migration and proliferation compared to contractile VSMCs. In addition, the development of hypertension has been found to be closely associated with the proliferation and migration of VSMCs [15]. Herein, our results corroborate these findings, demonstrating that Ang II treatment leads to upregulation of the synthetic protein OPN and downregulation of contractile proteins α-SMA and SM22α in VSMCs. Moreover, we found that Ang II treatment could significantly enhance VSMC migration and proliferation, and treatment with FOS notably reduced Ang II-induced phenotypic transformation, migration, and proliferation of VSMCs, validating the efficacy of the Ang II-induced hypertension *in vitro* model. Therefore, these results indicate that FOS can effectively prevent the Ang II-induced phenotypic transition, migration, and proliferation of VSMCs.

Oxidative stress is associated with maladaptive vascular remodeling, which can lead to hypertension or cardiovascular disease, and in this regard, reducing excess ROS is considered an effective strategy for treating oxidative stress-related cardiovascular conditions [16]. Research has demonstrated that Ang II activates NOX2 and NOX4, resulting in increased ROS levels, which in turn promote VSMC proliferation and migration [17]. Consistent with these findings, our results show that Ang II treatment significantly increased ROS levels, NAD(P)H activity, and the expression of NOX2 and NOX4 in VSMCs, confirming that Ang II induces oxidative stress. FOS administration effectively reversed these increases and mitigated Ang II-induced oxidative stress, indicating that FOS can inhibit oxidative stress in VSMCs induced by Ang II.

TGF-β1 is a key profibrotic factor among transforming growth factors, and Smad proteins serve as the primary intracellular effectors of TGF-β1 signaling, mediating fibrotic effects [18]. Ang II acts as an activator of TGF-β1, leading to increased TGF-β1 signaling through p-Smad2/3 [19]. It has been shown that tranilast can block the TGF-β1/Smad signaling pathway, thereby preventing Ang II-induced cardiac fibrosis [20]. One possible method by which FOS treats Ang II-induced kidney damage is by suppressing TGF-β1 expression [21]. In alignment with these findings, our results demonstrate that Ang II treatment led to upregulation of TGF-β1 and p-Smad2/3 and activation of the TGF-β1/Smad pathway in VSMCs. FOS administration significantly reversed these increases, indicating that FOS counteracts Ang II effects through inhibition of the TGF-β1/Smad pathway.

## 6 Conclusion

In conclusion, our present study elucidates the effects and mechanisms of FOS in regulating blood pressure and vascular remodeling *in vitro*. The results confirm that FOS inhibits Ang II-induced VSMC phenotypic transformation, proliferation, migration, and oxidative stress. Additionally, FOS exerts its anti-Ang II effects by targeting the TGF-β1/Smad signaling pathway. These findings support the potential of FOS as a treatment for hypertension. However, our study has certain limitations and further validation through co-culture and *in vivo* studies is needed.
